# Accessing Active Inference Theory through Its Implicit and Deliberative Practice in Human Organizations

**DOI:** 10.3390/e23111521

**Published:** 2021-11-15

**Authors:** Stephen Fox

**Affiliations:** VTT Technical Research Centre of Finland, FI-02150 Espoo, Finland; stephen.fox@vtt.fi; Tel.: +358-40-747-8801

**Keywords:** active inference, autoethnography, business models, environment, free energy principle, gap analysis, generative learning, joint agent-environment systems, process control, radar charts, survival, variational free energy

## Abstract

Active inference theory (AIT) is a corollary of the free-energy principle, which formalizes cognition of living system’s autopoietic organization. AIT comprises specialist terminology and mathematics used in theoretical neurobiology. Yet, active inference is common practice in human organizations, such as private companies, public institutions, and not-for-profits. Active inference encompasses three interrelated types of actions, which are carried out to minimize uncertainty about how organizations will survive. The three types of action are updating work beliefs, shifting work attention, and/or changing how work is performed. Accordingly, an alternative starting point for grasping active inference, rather than trying to understand AIT specialist terminology and mathematics, is to reflect upon lived experience. In other words, grasping active inference through autoethnographic research. In this short communication paper, accessing AIT through autoethnography is explained in terms of active inference in existing organizational practice (implicit active inference), new organizational methodologies that are informed by AIT (deliberative active inference), and combining implicit and deliberative active inference. In addition, these autoethnographic options for grasping AIT are related to generative learning.

## 1. Introduction

Many human organizations do not survive [1,2]. Apropos, it is recognized in organizational studies that better understanding is needed of interactions between inference, action, and learning [3,4,5]. Active inference theory (AIT) is relevant to this aim, as AIT encompasses interactions between inference, action, and learning at all levels of life from cells to societies [6]. In addition, AIT is applicable to automation technologies, which are becoming more widely used by human organizations [7].

However, AIT comprises specialist terminology and mathematics from theoretical neurobiology [8,9], which can obscure that active inference is an everyday experience in human organizations. In particular, active inference encompasses three interrelated types of actions, which can be carried out to minimize uncertainty about how organizations will survive. The three types of action are updating work beliefs, shifting work attention, and/or changing how work is performed. Accordingly, an alternative starting point for grasping active inference, rather than trying to understand AIT specialist terminology and mathematics, is to reflect upon lived experience. In other words, grasping active inference through qualitative autoethnographic research, which involves self-reflection on personal experience and connecting those reflections to wider contexts [10].

In this short communication paper, autoethnography is related to wider contexts through a common method in organizational practice: gap analysis with radar charts [11,12]. This is done to illustrate accessing AIT through common practice in human organizations. Next, in Section 2, an introduction to major constructs in active inference is provided in terms of radar charts. Then, in Section 3, a description is provided of active inference within established methods in organizational practice (i.e., implicit active inference). Subsequently, in Section 4, combination of implicit and deliberative active inference is discussed, with deliberative active inference being explicit application of AIT in the development of organizational practice. In conclusion, in Section 5, generative learning of AIT is related to autoethnography involving reference to active inference practice in human organizations.

## 2. Active Inference Constructs

### 2.1. Varying Survival Information Deficit (i.e., Variational Free Energy)

Living things aim to minimize their uncertainty about how they will survive in the environments in which they are situated. Uncertainty comprises information gaps between living things’ models of themselves in the environment and their actual selves in the real environment, for example, an information gap between an organization’s sales forecast and its actual sales (e.g., forecast sales of 120 products per week versus actual sales of 100 products per week). One information gap can be related to another. For example, sales forecasts and actual sales are related to cash flow forecasts and actual cash flow (e.g., 120,000 euros income per week versus 100,000 euros income per week). A technical term used in theoretical neurobiology for such information gaps is surprisal.

**Figure 1:**
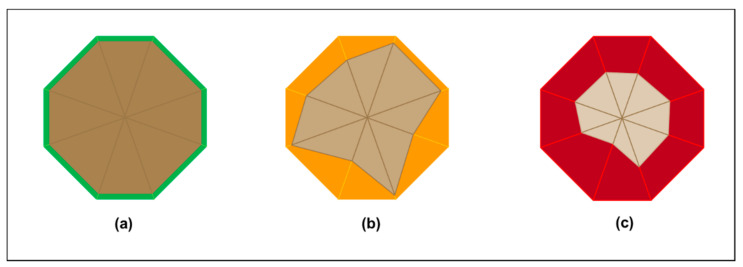
Varying survival information deficit. (**a**) Organization (inner shape) has good fit with the environment (outer shape) due to minimal survival information deficit (green): organization can prosper in the environment. (**b**) Survival information deficit increases but is tolerable (orange): organization can survive in the environment. (**c**) Survival information deficit increases further and is not sustainable (red): organization cannot survive in the environment unless it improves its fit with the environment. The red area illustrates the maximum survival information deficit for the organization in its current environment.

As illustrated in Figure 1, which uses the radar chart gap analysis format, information gaps combine in the survival information deficit. This varies as living things change and as environments change. In neurobiology terminology, the varying survival information deficit can be described as variational free energy. The bigger the survival information deficit, the worse the fit with the environment. As illustrated in Figure 1c, there is an upper limit to survival information deficit beyond which survival is not possible. In neurobiology terminology, this maximum survival information deficit can be described as variational free energy upper bound [13,14].

As with other living things, different human organizations have different maximum survival information deficits at different times as they change and as environments change. For example, an organization with large cash reserves can survive the information gap (i.e., surprisal) of slightly fewer sales than it forecast. Subsequently, however, when the company has used up all its cash reserves, the same company may not be able to survive lower sales than it has forecast because its financial outgoings (i.e., costs) will be more than its financial incomings (i.e., revenue).

### 2.2. Forecasting Errors (i.e., Prediction Errors)

**Figure 2:**
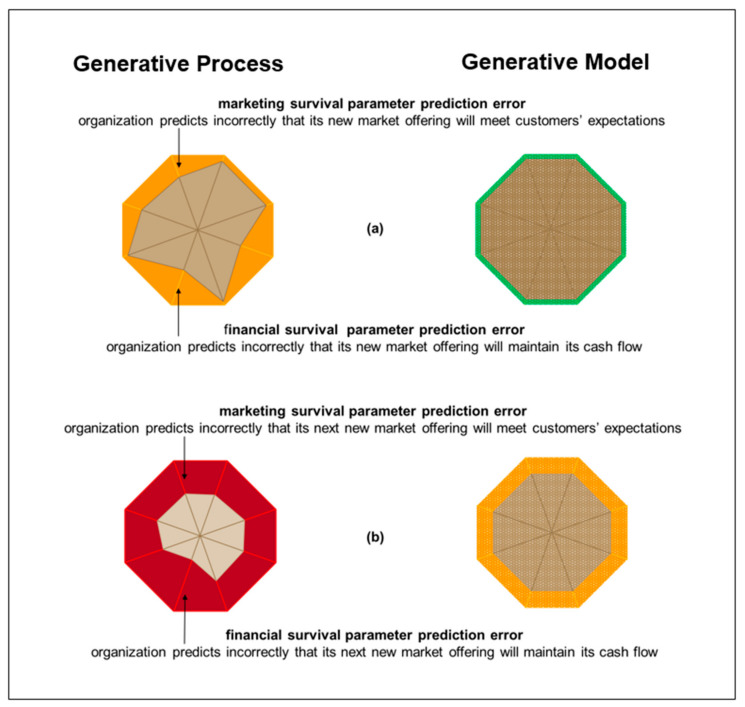
Forecasting errors. (**a**) Expected minimal survival information deficit (green) in the organization’s generative model of itself in its environment is increased by forecasting errors about survival parameters to tolerable survival information deficit (orange). (**b**) Expected tolerable survival information deficit (orange) in the organization’s generative model of itself is increased by forecasting errors on survival parameters to unsustainable survival information deficit (red).

As summarized in Figure 2, in order to reduce survival information deficit, organizations can take actions along numerous parameters to change themselves and/or the environments in which they are situated. From an ecological perspective, these survival parameters can be described as fitness components [15]. The term generative process refers to the actual structure of the world that generates observations that are made by the organization. Generative model refers to how the organization expects the observations to be generated [14]. In practice, a generative model corresponds to a business model [16].

Organizations’ actions are based on their forecasts about the effects of their actions on their survival parameter information gaps. These parameters can include, for example, marketing, product development, procurement, production, delivery, finance, etc. Together, parameters can comprise an organization’s business model [16], which can be described as a generative model in the context of theoretical neurobiology terminology. Forecasts are statements of survival preferences. For example, a preference for surviving in competitive markets through high volume sales of economy goods. Forecasts are predictions of future observations rather than plans that will be inevitably realized. This is because organizations cannot know all causal factors in the environment that can affect their preferred observations, such as preferred observations of high-volume sales. Rather, many causal factors are hidden from the organization. They can be hidden because of the general difficulty of acquiring perfect information. In addition, they may be hidden because of deliberate concealment, such as competitors keeping secret their rapid development of new products. Apropos, in the context of theoretical neurobiology terminology, factors that contribute to causing observations are referred to as hidden states.

Actions are taken iteratively to reduce differences between what is forecast to be observed and what is observed (i.e., to reduce prediction errors). Actions can involve making use of existing information (i.e., exploiting existing information) and/or searching for other information (i.e., exploring for new information). Attention and work can be based more on internal beliefs than information coming from the environment if an organization has more confidence in internal beliefs than in information coming from the environment; or vice versa. Actions can be updating beliefs about how to survive, shifting focus of attention in trying to survive, and/or changing work performed in order to survive. Actions can involve changing self and/or environment in order to improve fit between self and environment.

Figure 2a illustrates an organization making forecasting errors in marketing and in finance. In particular, the organization forecasts incorrectly that its new market offering will meet customers’ expectations, and that sales from its new market offering will maintain its cash flow. Consequently, its actual survival information deficit is more than its expected survival information deficit. Figure 2b illustrates that the organization does update its generative model so that its expected survival information deficit is larger but tolerable. However, the organization does not take the needed action of shifting its attention to pay closer attention to customers’ expectations. In addition, the organization does not take the needed action of changing its work to provide a new market offering that better meets customers’ expectations. Hence, the organization’s actual survival information deficit in the generative process of the environment is more than its expected survival information deficit and increases beyond tolerable to unsustainable.

### 2.3. Organizational Identity (i.e., Expected Free Energy)

As illustrated in Figure 2a, forecasting errors can combine to increase actual survival information deficit. Then, as illustrated in Figure 2b, actual survival information deficit can lead to an organization updating its expected survival information deficit. In the context of theoretical neurobiology terminology, expected survival information deficit can be described as expected free energy. It is important to note that while forecasts can be made for survival parameter information gaps, forecasts cannot be made for expected survival information deficit.

In the context of theoretical neurobiology, explanations of why expected free energy cannot be predicted involve specialist mathematics. By contrast, those working in organizational practice can make reference to everyday experiences to understand why expected survival information deficit cannot be forecast. For example, from an internal perspective, different survival parameters involve different types of information, such as product sales in product units and cash flow in financial currency. Accordingly, survival information gaps cannot be added together into an overall amount. From an external perspective, market responses are different on different survival parameters. For example, competitors may introduce new products and banks may change financial arrangements. Again, these changes cannot be added together into an overall amount. Moreover, there can be unpredictable interactions between different external organizations and other causal factors in the environment that are hidden from the organization. For example, sudden weather events can disrupt both supply into the organization and demand for its products.

Hence, rather than being a forecast, expected survival information deficit is an expectation of the impression that an organization will make on its environment. This can be considered in terms of organizational identity, which encompasses how an organization prefers to see itself and would prefer to be seen by others [17,18]. For example, an organization could prefer to be seen as an organization with a strong financial base that provides its customers with good value products. As observations of an organization will be different among different people, expected survival information deficit can be considered as an average.

## 3. Implicit Organizational Active Inference

**Figure 3:**
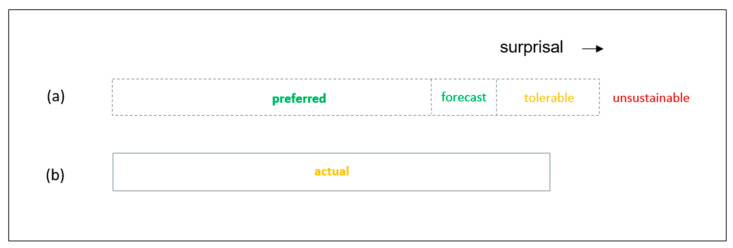
Gantt chart style activity bar. (**a**) The planning bar shows the preferred, forecast, tolerable, unsustainable ranges for the activity. The term surprisal is applicable to the tolerable range and unsustainable range. (**b**) The bar shows the actual activity outcome, which is more than expected but within tolerable limits.

Active inference is implicit in some existing practices in human organizations. In this section, a short summary is provided that goes beyond extant literature [19] by relating process management charts to AIT surprisal and to countervailing preferences for suprisal minimization within human organizations [20]. As shown in Figure 3, organizations can plan and monitor survival parameter actions using bar charts, such as Gantt charts [21]. These charts are widely used in project activities, such as product development. Figure 3a illustrates that the bars on such charts can provide summaries of preferred, expected, tolerable, and unsustainable outcomes from actions. The forecast is that the action outcome will be within the expected range. There is a forecasting error because, as shown in Figure 3b, the actual action outcome is outside the expected range on the survival parameter.

**Figure 4:**
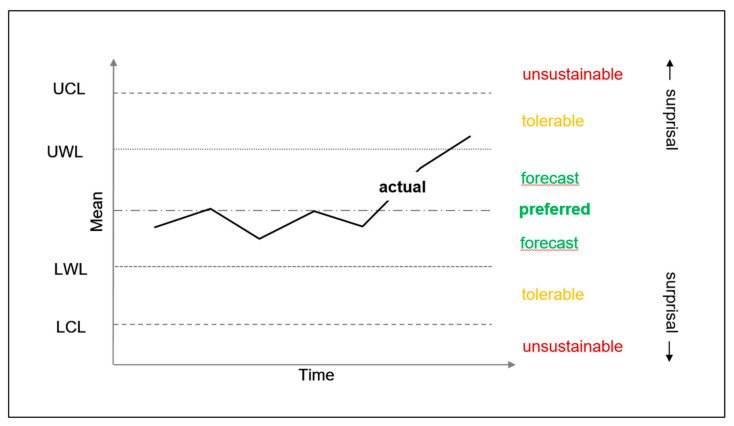
Statistical process control chart. The forecast is that the process will stay within the expected deviation from the mean. UWL (upper warning limit) and LWL (lower warning limit) indicate the limits of expected deviation from the mean that is the preferred process outcome. UCL (upper control limit) and LCL (lower control limit) indicate the limit of tolerable deviation from the mean. The actual processes have been operating within the expected deviation from the mean (i.e., between UWL and LWL) but have drifted outside the expected range. Thus, there is a forecasting error. The term surprisal is applicable to the tolerable range and unsustainable range.

As shown in Figure 4 below, organizations can also plan and monitor survival parameter actions using statistical process control charts [22]. These charts are widely used in repetitive activities, for example in mass production. Figure 4 shows that such charts can have upper control limits (UCL) and lower control limits (LCL), within which are upper warning limits (UWL) and lower warning limits (LWL). UWL and LWL indicate the limits of expected deviation from the mean that represents the preferred process outcome. UCL and LCL indicate the limit of tolerable deviation from the mean. The forecast is that the process will stay within the expected deviation from the mean, i.e., between the UWL and the LWL. In Figure 4, the actual processes have been operating within the expected deviation from the mean (i.e., between UWL and LWL) but have begun to drift outside the expected range. Thus, there is a forecasting error.

It is routine for organizations to make precise forecasts about survival parameter actions. These can include sales forecasts for a new market offering and forecasts of cash flows from those sales (Figure 2). They can also include durations for product development projects that are intended to bring new products to market before competitors (Figure 3). In addition, they can include manufacturing dimensions for new product parts (Figure 4). As illustrated in Figure 2, predictions errors on individual survival parameters are interrelated in affecting survival information deficit. However, their interactions are characterized by dynamic complexity between each other and as they interact with the environment. For example, launching a new product later than predicted during product development planning can give competitors an advantage. Also, manufacturing outside of predicted dimensions can lead to poor product operation, which leads to potential customers buying competitors’ products. Both of these can lead to lower than predicted sales and lower than predicted cash flow. However, effects on sales and cash flow are also dependent upon the actions of competitors. Thus, survival information deficit cannot be predicted precisely. Rather, process charts are applied to individual internal processes on individual survival parameters. They do not enable organizations to control the environment, which can exert determining influence over survival information deficit. Hence, predictions about the outcome of an action, or a series of actions, on a survival parameter are not predictions of survival information deficit. Rather, they provide a basis for expectations about the survival information deficit.

Within active inference theory (AIT), there are not process management constructs such as those in Gantt charts and SPC charts. Rather, there are preferred observations and the fundamental goal of surprisal minimization. Accordingly, there are not gradations such as tolerable and unsustainable in AIT. However, organizations need process management charts in order to manage countervailing preferences within organizational silos [20]. For example, a product development department may prefer to spend more money and take more time than competitors to minimize product surprisal. In other words, to minimize the gap between the product to be offered by the organization and potential customers’ ideal product. By contrast, the sales department would prefer to introduce a new product at a lower price and before its competitors. This being preferred to minimize sales surprisal, i.e., to minimize the gap between sales forecasts and actual sales. Apropos, process management charts are used to address the potential for organizations’ personnel to minimize the source of surprisal that is most important to them, but to increase another source of surprisal that is less important to them. In addition, process management charts address the potential for causes beyond the control of the organization to affect processes. For example, SPC charts used in manufacturing address the potential for the properties of natural materials to affect the accuracy of their machining. Consider, for example, the potential for knots in hardwood to decrease the precision of their machining into furniture components. This can lead to machining drifting above warning limits towards control limits. This can happen despite the best efforts of personnel to keep machining within warning limits: i.e., to minimize machining surprisal. Here, there can again be countervailing preferences that need to be managed. For example, machining surprisal could be minimized by the organization buying hardwood that has far fewer knots. However, such hardwood could be far above the financial budget for manufacturing the furniture components. Thus, machining surprisal could be minimized at the expense of budget surprisal. So it is that minimizing surprisal in human organizations can involve managing multiple countervailing preferences with the aid of process management charts.

## 4. Combining Implicit and Deliberative Organizational Active Inference

Deliberative active inference involves making reference to AIT literature in the development of new organizational practice. One detailed example of deliberative active inference is reported in [23]. As summarized in Table 1 below, there can be five generalizable characteristics of deliberative active inference. First, there is an aspect of organizational survival that is not addressed successfully already by implicit organizational active inference. Second, AIT literature suggests new directions for addressing the issue. Third, AIT can be related to other theoretical resources from research in the natural sciences. Fourth, new methods based on deliberative active inference can be related to established constructs from organizational studies. Fifth, new methods based on deliberative active inference can be related to established organizational practice. The remainder of this section describes a new example in which these five characteristics are considered, and in which there can be combination with implicit active inference from the outset. In particular, implicit active inference in the use of radar charts [11,12].

As summarized in Table 1, an aspect of organizational survival that is not addressed successfully by implicit organizational active inference is the fit of organizations with the environments in which they intended to survive and grow. In particular, fit is not addressed in terms of the reciprocal synchronicity of organizations and environments as they jointly learn to adapt to each other into order to co-exist successfully. AIT literature suggests new directions for addressing this issue through studies concerned with joint agent-environment systems [14] and model structure learning [24]. AIT can be related to other theoretical resources from research in the natural sciences concerned with joint learning and development of agents and environments [25]. New methods based on deliberative active inference can be related to established constructs from organizational studies, for example, organizational lifecycles [26] and organizational design [27]. New methods based on deliberative active inference can be related to established organizational practice in business model design [16] and set-based design [28].

In order to survive, organizations need to adapt their business models, which set-out their what (e.g., market offerings), their how (e.g., operating processes) and their why (e.g., value proposition). Here, it is important to note that notion of business models being generative models is established in organizational studies. For example, it has been argued that business models generate virtuous cycles, or feedback loops, that are self-reinforcing [16]. However, this notion does not address the fundamental problem that self-reinforcement of a business model can lead an organization not learning sufficiently about environmental changes in order to undertake necessary business model adaptation. For example, it is possible for a global provider of instant photographs to recognize that its technology would be superseded by digital photography. Nonetheless, failure to carry out work to adapt in order to provide digital photography can lead to the organization not surviving [29]. More generally, an organization may not survive if its attention and its work are based more on its out-of-date internal beliefs than on new information coming from the environment [30,31]. This fundamental problem is addressed directly in studies concerned with joint agent–environment systems, for example, with formulations such as a stubborn agent might persist in its behavior despite contrary evidence [14]. From an organizational perspective, contrary evidence could be, for example, falling product sales, decreasing revenues, cash flow problems, and other contractions that can lead an organization into a so-called death spiral [32].

AIT literature concerned with model structure learning provides important insights into how organizations can keep themselves open to learning from the environment, rather than learning only to reinforce their existing internal model. In particular, AIT literature encompasses a generative model being equipped with open slots for learning about new concepts. These open slots facilitate model expansion, which can be followed by model reduction in order to prevent models becoming overly complex [24]. Similar in organizational practice is set-based design. This is an approach to design that involves being open to multiple design options simultaneously, rather than successively criticizing and modifying a single design option [28]. However, set-based design is focused on product development and business model design can be focused on reinforcement. Apropos, changing business models from one to another often involves the abrupt change of so-called pivoting [33] rather than continual synchronous adaptation with the environment. By contrast, reference to AIT literature highlights the need for continuous learning with the environment [14] and the need to keep open slots for model expansion together with procedures preventing models from becoming overly complex [24]. Thus, reference to AIT stimulates consideration of set-based business model design in practice in accordance with fundamental active inference questions: should we change our work, should we shift our attention, and/or should we update our current generative model in accordance with new learning from the environment. Throughout, radar charts can be used to regularly map both the organization and the environment as shown in Figure 1 and Figure 2 above.

## 5. Conclusions

Theoretical neurobiology terminology and mathematics used in AIT may not be the best starting points for explaining AIT to the widest range of potential beneficiaries. An alternative is for explanation to begin with individuals engaging in autoethnography, i.e., self-reflection on personal experience and connecting reflections to wider contexts [10]. This may be a better alternative for many because all living things that survive are already practitioners in what is intended to be described by AIT terminology and mathematics. In particular, AIT is concerned with the daily experiences of trying to minimize uncertainty about how to survive by updating beliefs, shifting attention, and performing work, which may or may not be influenced by information from the environment depending upon confidence in beliefs. Thus, understanding of active inference can begin by reflecting upon lived experience, such as poor marketing leading to cash flow problems that undermine organizational survival (Figure 2). Then, that lived experience can inform selection and application of AIT constructs when seeking better explanation and improvement of efforts to survive amidst changing environments.

Autoethnography can involve consideration of implicit and deliberative active inference in organizational practice using common methods such as radar charts. Importantly, relating AIT to widely used methods such as radar charts has potential to facilitate generative learning of AIT by people who do not have a background in theoretical neurobiology. Generative learning is the process of transforming incoming information into usable knowledge. Generative learning involves actively constructing meaning from to-be-learned information by mentally reorganizing it and integrating it with one’s existing knowledge, for example, existing knowledge of methods applied in organizational practice. Within generative learning, the mind is not a passive consumer of information. Rather, the mind actively constructs its own interpretations of information and draws inferences from those interpretations. This form of active cognitive processing enables learners to develop an understanding of incoming information that they can apply in new situations [34]. For some, the specialist AIT terminology and mathematics used in theoretical neurobiology may provide the best format of incoming knowledge to transform into usable knowledge. For many others, charts and other methods used in everyday organizational practice may provide a better starting point.

## Figures and Tables

**Table 1:** Characteristics of Deliberative Active Inference.

Characteristic	Example
Issue in organizational survival not addressed successfully by implicit active inference in extant methodologies	Reciprocal synchronicity of organizations and their environments
AIT literature suggests new directions for addressing the organizational issue	AIT studies concerned with joint agent-environment systems
AIT can be related to other theoretical resources from natural science research	Natural science research concerned with joint learning and development of agents and environments
New deliberative active inference method can be related to established constructs from organizational studies	Organizational lifecycles and organizational design
New deliberative active inference method can be related to established organizational practice	Business model design and set-based design

## Data Availability

Not applicable.
